# Therapeutic Effects of Natural Drugs on Alzheimer’s Disease

**DOI:** 10.3389/fphar.2019.01355

**Published:** 2019-12-04

**Authors:** Yuan Ma, Man-wen Yang, Xin-wei Li, Jian-wei Yue, Jun-zong Chen, Mei-wen Yang, Xuan Huang, Lian-lian Zhu, Fen-fang Hong, Shu-long Yang

**Affiliations:** ^1^Department of Physiology, College of Medicine, Nanchang University, Nanchang, China; ^2^Department of Nurse, Nanchang University Hospital, Nanchang, China; ^3^Department of Experimental Teaching Center, Nanchang University, Nanchang, China

**Keywords:** Alzheimer's disease, β-amyloid, tau protein metabolism, free radical damage, natural drug

## Abstract

Alzheimer disease (AD) is characterized as a chronic neurodegenerative disease associated with aging. The clinical manifestations of AD include latent episodes of memory and cognitive impairment, psychiatric symptoms and behavioral disorders, as well as limited activities in daily life. In developed countries, AD is now acknowledged as the third leading cause of death, following cardiovascular disease and cancer. The pathogenesis and mechanism of AD remain unclear, although some theories have been proposed to explain AD, such as the theory of β-amyloid, the theory of the abnormal metabolism of tau protein, the theory of free radical damage, the theory of the inflammatory response, the theory of cholinergic damage, etc. Effective methods to predict, prevent or reverse AD are unavailable, and thus the development of new, efficient therapeutic drugs has become a current research hot spot worldwide. The isolation and extraction of active components from natural drugs have great potential in treating AD. These drugs possess the advantages of multiple targets in multiple pathways, fewer side effects and a long duration of curative effects. This article summaries the latest research progress regarding the mechanisms of natural drugs in the treatment of AD, providing a review of the literature and a theoretical basis for improving the clinical treatment of AD.

## Introduction

Alzheimer disease (AD) is characterized as a chronic neurodegenerative disease associated with aging. The clinical manifestations of AD include latent episodes of memory and cognitive impairment, psychiatric symptoms and behavioral disorders, as well as limited activities in daily life (Sun et al., 2013). As the global population ages, dementia has become an important health problem worldwide. It is estimated that the global prevalence of dementia will increase to 81.1 million in 2040 from 24.3 million in 2005 (Chua et al., 2015). In addition, AD is the most common type of dementia, accounting for 60% of all dementia cases. In developed countries, AD is now considered the third main cause of death, after only cardiovascular disease and cancer (Huang et al., 2016). AD usually has a late onset, and its prevalence will double every 5 years after age 65 (Ballard et al., 2011). AD is caused by genetic and environmental factors. Genetic factors account for approximately 70% of cases and are closely related to three variants of the apolipoprotein E (APOE) gene (*ε2*, *ε3*, and *ε4*) (Verghese et al., 2011). Other diseases, such as hypertension, diabetes, and obesity, can increase the risk of AD to different extents (Xu et al., 2015). The pathogenesis and mechanism of AD remain unclear. However, many pathological features are known. The most important pathological features are amyloid plaques and neurofibrillary tangles, which form following the aggregation of β-amyloid (Aβ) proteins and hyperphosphorylated tau protein, respectively. In addition, other pathological features include cell damage caused by oxidative stress, neuroinflammation and injury to cholinergic neurons, which cause neurodegeneration and a loss of synapses and neurons, leading to macroscopic atrophy of the brain regions associated with learning and memory, including the temporal and frontal areas and the hippocampus (Ryu et al., 2019). Therefore, current research mainly focuses on five theories, namely, the β-amyloid theory, abnormal tau protein metabolism theory, free radical damage theory, inflammatory response theory and cholinergic damage theory. All of these theories provide a basis for improving our understanding and research on AD. Currently, the main clinical treatment for AD is acetyl cholinesterase inhibitors (AChEIs) or antagonists of N-methyl-D-aspartate receptor (NMDAR). AChEIs, such as donepezil, galantamine and rivastigmine, are effective in patients with mild and moderate symptoms. These compounds can inhibit acetylcholine (ACh) degradation in the synapse to increase its availability (Birks, 2006). Antagonists of NMDAR, such as memantine, are effective at treating moderate and severe symptoms in patients. These compounds can reduce the excitatory neurotoxicity of L-glutamate without inhibiting its physiological effects (McShane et al., 2006). Based on accumulating evidence, both types of drug improve patients' behavioral and cognitive impairments, but these clinical drug treatments only relieve the symptoms of the disease and do not prevent progression (Huang and Mucke, 2012; Cheung et al., 2015). Thus, the development of new, effective drugs for treating AD has become a global focus. In this review, we have focused on the potential therapeutic effects of natural drug extracts on AD and solved all the taxonomic ambiguities and errors in original papers (Rivera et al., 2014). Our objective is to assess the accuracy of plants scientific nomenclature and to explore the mechanisms by which these natural drug extracts regulate AD.

### Treatments Targeting Aβ

As one of the main features of AD, the Aβ protein is the main component of senile plaques. The accumulation of the Aβ protein is the principal pathological factor of AD and is considered the driving force by some scholars (Moon et al., 2014), becoming the most significant target in the prevention and control of AD.

#### Inhibition of Aβ Formation

Under normal conditions, the β-amyloid precursor protein (APP) is cleaved by α-secretase and γ-secretase, and no Aβ is formed. If the APP gene is mutated, β-secretase and γ-secretase cleave APP to increase Aβ production. Thus, a treatment that selectively increases the activity of α-secretase and decreases the activity of β-secretase will help reduce the formation of Aβ, subsequently slowing the progression of AD. Among these enzymes, β-secretase is the rate-limiting enzyme that catalyzes the production of the Aβ peptide and is a key factor in the pathogenesis of AD. Natural drug products have been used to treat AD for many years, and some extracts inhibit enzymes required for Aβ production. The natural products of traditional Chinese medicine (TCM), which are widely used in folk medicine, display superior safety (Zhang, 2012).

**Figure 1:**
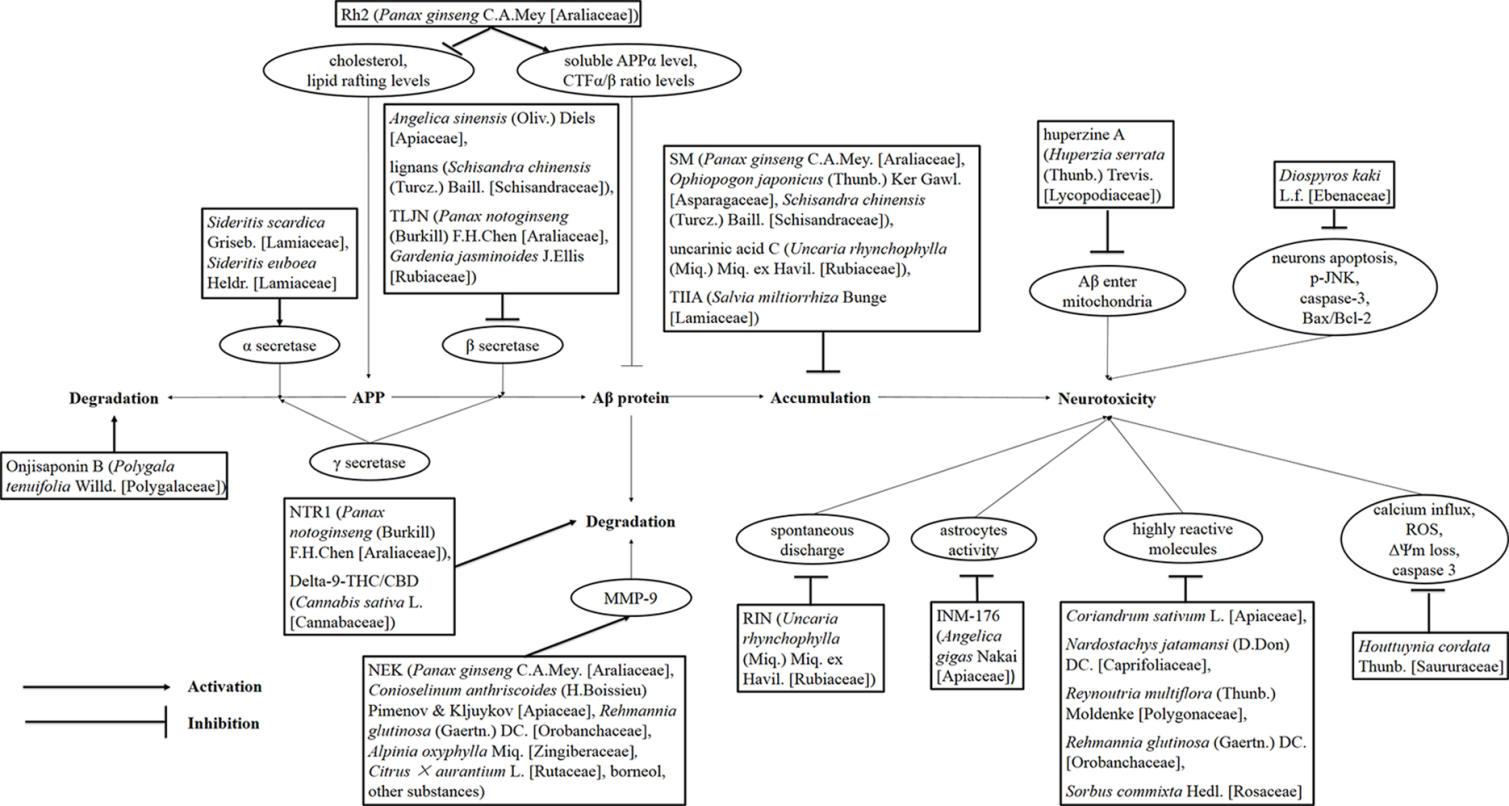
Drug treatment of AD induced by Aβ. Rh2, ginsenoside Rh2; TLJN, TongLuoJiuNao; SM, Shengmai; TIIA, tanshinone IIA; JNK, c-Jun N-terminal kinase; NTR1, notoginsenoside R1; THC, tetrahydrocannabinol; CBD, cannabidiol; NEK, Naoerkang; MMP, matrix metalloproteinase; RIN, rhynchophylline; INM-176, a standardized ethanolic extract of *Angelica gigas* Nakai (Apiaceae).

**Figure 2:**
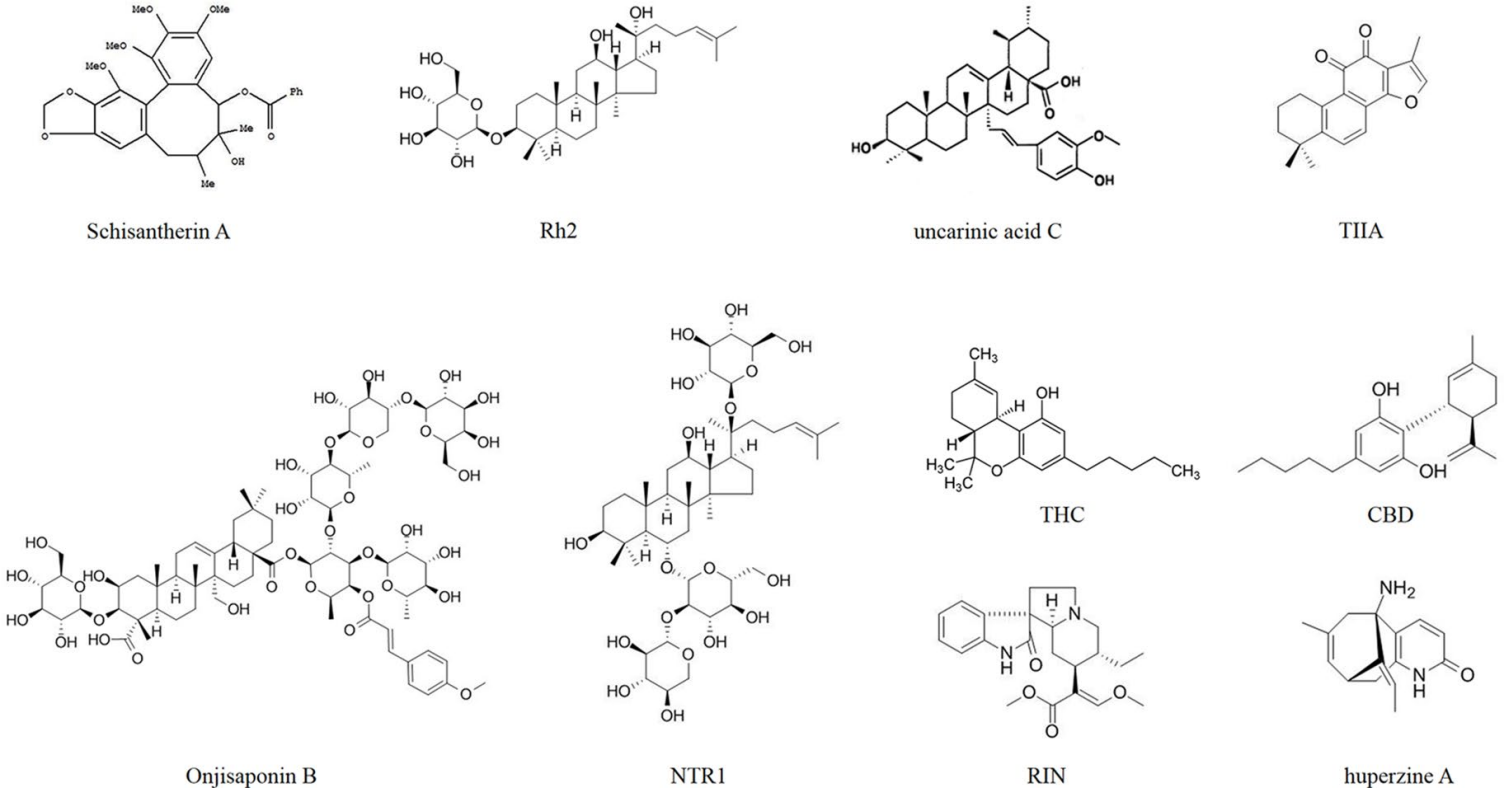
Chemical structures of some of the drugs used to treat Aβ.

Some researchers injected AD model mice with Aβ and then administered natural drug extracts, achieving considerable results. As shown in the study by Duan et al. (2016), the administration of *Angelica sinensis* (Oliv.) Diels (Apiaceae) extracts to rats targets β-secretase in the hippocampus and is associated with reduced Aβ levels according to western blots (**Figure 1**). Jeong et al. (2013) orally administered schisantherin A [*Schisandra chinensis* (Turcz.) Baill. (Schisandraceae) extracts] to a model mouse strain and observed reduced β-secretase activity and Aβ1-42 levels in the cerebral cortex and hippocampus (**Figure 2**). According to Qiu et al. (2014), ginsenoside Rh2 (Rh2) extracted from *Panax ginseng* C.A.Mey. (Araliaceae) (root) increases the soluble APPα level and carboxyl terminal fragment (CTF) α/β ratio in hippocampal neurons, and decreases the concentrations of Aβ40 and Aβ42 when Rh2 is injected into Tg 2576 AD model mice. In addition, Rh2 regulates APP cleavage by decreasing cholesterol levels and the number of lipid rafts, reducing the formation of senile plaques in the brain of mice at this age. The memory impairment of AD model mice was improved by the treatment, and the Morris water maze test showed that the memory and behavior impairment of AD model mice were even reversed by the treatment.

On the other hand, APP transgenic mice and nontransgenic aging C57BL/6 mice were orally administered *Sideritis scardica* Griseb. (Lamiaceae) and *Sideritis euboea* Heldr. (Lamiaceae) extracts, and the expression of the α-secretase A Desintegrin and Metalloproteinase (ADAM) 10 increased, and the deposition of Aβ42 in APP transgenic mice was substantially reduced. The cognitive ability of elderly, nontransgenic and APP transgenic mice was substantially increased. Compared with the nontransgenic control group, the treatment completely reversed the neuronal loss observed in the APP transgenic mice and even restored the normal number of neurons (Hofrichter et al., 2016). He et al. (2013) chronically administered 6-month-old APP mutant transgenic mice (APP23) the herbal medicine TongLuoJiuNao [TLJN, extracted from *Panax notoginseng* (Burkill) F.H.Chen (Araliaceae) and *Gardenia jasminoides* J.Ellis (Rubiaceae) follow by processed and purified in accordance with the protocol of the National Medical Dictionary of China] found that TLJN can decrease the level and activity of β-secretase and the expression of components of the β-secretase complex, significantly reducing the production and deposition of Aβ in the brains of APP23 mice.

#### Inhibition of Aβ Accumulation

Zhang et al. (2016b) used transgenic *Caenorhabditis elegans* as a model of AD at the original ecological level and found that the Shengmai (SM) formula water decoction [*P. ginseng* C.A.Mey. (Araliaceae): *Ophiopogon japonicus* (Thunb.) Ker Gawl. (Asparagaceae): *S. chinensis* (Turcz.) Baill. (Schisandraceae)= 9:9:6 (SM), 9:18:9 (SM1), 2:2:1 (SM2), 3:2:1 (SM3), 9:0:0 (SM4), 0:9:0 (SM5), 0:0:6, 9:9:0, 9:0:6, 0:9:6, respectively] and ethanol extract (9:9:6) could improve the paralytic behavior and the pathological characteristics of chemotaxis defects in the transgenic *Caenorhabditis* by reducing the accumulation of Aβ and inhibiting the expression of the homologous genes hsp16-2, hsp16-41, ace-1, ace-2, TNFA1P1. Based on these results, SM powder has great potential as a treatment for AD. According to Yoshioka et al. (2016), uncarinic acid C from *Uncaria rhynchophylla* (Miq.) Miq. ex Havil. (Rubiaceae) is a specific inhibitor of the nucleation stage of Aβ42 aggregation. Structure–activity studies revealed that c-27 ferulic acid and c-28 carboxylic acid groups inhibit the activity of related enzymes. Other researchers (Li et al., 2016a) intraperitoneally injected postnatal mice with 25–100 mg/kg tanshinone IIA (TIIA) extracted from the root of *Salvia miltiorrhiza* Bunge (Lamiaceae) for 30 consecutive days and found that TIIA could reduce the accumulation of Aβ1-42 and CTFs in the hippocampus of the AD model mice. Moreover, the memory impairment in AD mice can be alleviated after treatment with TIIA at doses of 50 and 100 mg/kg.

#### Promotion of Aβ Degradation

In the APP/Presenilin-1 (PS1) double-transgenic AD model mice, Li et al. (2016b) found that Onjisaponin B, one of active constituent of *Polygala tenuifolia* Willd. (Polygalaceae), inhibited Aβ production without directly inhibiting the activation of β-site amyloid precursor protein cleaving enzyme 1 (BACE1) and γ-secretase but promoted the degradation of APP to ameliorate Aβ pathology and behavioral defects in a mouse model. Li et al. (2015b) administered 5 or 25 mg/(kg·day) notoginsenoside R1 [NTR1, extracted from *P. notoginseng* (Burkill) F.H.Chen (Araliaceae)] or vehicle *via* oral garage to 3-month-old APP/PS1 mice for 3 months. The therapeutic effects on the two groups were compared, and the cognitive function of the NTR1 group was significantly improved. The NTR1 treatment can inhibit the accumulation of Aβ and the expression of insulin-degrading enzyme in APP/PS1 mice and n2a-app695sw cells, suggesting that NTR1 may exert its protective effects by the enhancement of Aβ production (Li et al., 2015b).

Aso et al. (2015) administered delta-9-tetrahydrocannabinol (THC)/cannabidiol (CBD), two phytocannabinoids produced by the plant *Cannabis sativa* L. (Cannabaceae) to APP/PS1 mice and observed a significant decrease in soluble Aβ42 peptide levels and a change in the plaque composition. Moreover, the administration of the drug at the early symptomatic stage could maintain the memory of the transgenic mice, indicating that the harmful effect of Aβ peptide can be reduced by THC/CBD.

Researchers divided AD model rats into normal control, untreated, piracetam, and Naoerkang (NEK) groups to systematically study the therapeutic effect of NEK on AD model rats. NEK is composed of *P. ginseng* C.A.Mey. (Araliaceae), *Conioselinum anthriscoides* (H.Boissieu) Pimenov and Kljuykov (Apiaceae) (rhizome), *Rehmannia glutinosa* (Gaertn.) DC. (Orobanchaceae) (rhizome), *Alpinia oxyphylla* Miq. (Zingiberaceae), *Citrus × aurantium* L. (Rutaceae) (fructus), borneol and other substances. The rats in the NEK groups were treated with a high [60 g/(kg·day)], medium [30 g/(kg·day)] or low dose [15 g/(kg·day)] of NEK. Piracetam (0.375 g/kg, intragastrically) was consecutively administered to the piracetam group. The normal control and untreated groups were treated with the same dose of normal saline. Twenty-eight days after the administration of the different treatments, the learning and memory abilities of AD model rats in the piracetam and NEK groups were significantly improved, the levels of Aβ1-42 in the hippocampus decreased, and matrix metalloproteinase-9 (MMP-9) levels increased compared with the untreated group. Thus, NEK may exert its anti-dementia effect by increasing the expression of MMP-9 and reducing Aβ1-42 production (Li et al., 2011b).

#### Inhibition of Aβ Neurotoxicity

Some scholars who support Aβ as the driving factor of AD pathogenesis insist that the gradual deposition of Aβ in the cerebral cortex and hippocampal neurons causes a series of cross-cascade reactions, such as glial cell inflammation, oxidative stress, disruption of calcium homeostasis, synaptic dysfunction and loss of a large number of neurons. These changes would ultimately lead to brain atrophy and the destruction of neural structure and function. Recently, a study showed that soluble Aβ1-42 oligomers induced hyperactivity and perturbed the firing patterns of hippocampal neurons. In follow-up studies, Shao et al. (2015) found that treatment with rhynchophylline(RIN) effectively inhibited the soluble Aβ1-42 oligomer-induced increase in spontaneous discharges in a concentration-dependent manner (IC50=9.0 µM). RIN is an active tetracyclic oxindole alkaloid isolated from *U. rhynchophylla* (Miq.) Miq. ex Havil. (Rubiaceae). It also counteracted the harmful effects of Aβ1-42 on the neural circuits.

Microglia are the main immunocompetent phagocytes in AD and play an important role in regulating neuroinflammatory plaques and neurofibrillary tangles. The overactivation of microglia may produce direct toxic effects on neurons (Huayan et al., 2014). In the AD rat model induced by Aβ, Park et al. (2012) found that INM-176 [ethanolic extract of *Angelica gigas* Nakai (Apiaceae)] could decrease the activation of astrocytes and the damage of cholinergic neurons in the hippocampal CA3 region and basal ganglia of Meynert that ameliorated memory impairments in model mice. Aβ causes the excessive production of highly reactive molecules, such as reactive oxygen species (ROS), through multiple pathways, which overwhelms the antioxidant capacity and leads to oxidative stress in the brain. Liu et al. (2015) established a rapid *in vivo* screening system using *Drosophila* AD models. Among the 23 medicinal plants tested, the extracts from five plants, *Coriandrum sativum* L. (Apiaceae), *Nardostachys jatamansi* (D.Don) DC. (Caprifoliaceae), *Reynoutria multiflora* (Thunb.) Moldenke (Polygonaceae), *R. glutinosa* (Gaertn.) DC. (Orobanchaceae) and *Sorbus commixta* Hedl. (Rosaceae) exerted protective effects on Aβ42 neurotoxicity. *R. multiflora* (Thunb.) Moldenke (Polygonaceae) or *S. commixta* Hedl. (Rosaceae) extracts were fed to flies expressing Aβ42 with AD-related neurological phenotypes (e.g., decreased survival and motility and increased cell death and reactive oxygen species levels), and the results showed strong neural inhibition. Furthermore, the main component of *R. multiflora* (Thunb.) Moldenke (Polygonaceae), 2,3,5,4-toluylene-2-O-β-D-glucoside, exerts a similar protective effect on human cells exposed to Aβ42 cytotoxicity. The accumulation of Aβ on the mitochondrial membrane will destroy the mitochondrial membrane potential, which disrupts the electron transport chain and oxidative phosphorylation. Then, excessive ROS are produced in neurons, causing oxidative stress and damaging the normal structure and function of the mitochondria. Finally, the energy metabolism becomes abnormal. Meanwhile, the activation of mitochondrial apoptosis-related proteins can initiate the mitochondrial apoptosis pathway in neurons (Yang and Liu, 2012). Park and Oh (2012) pretreated neurons with *Houttuynia cordata* Thunb. (Saururaceae) water extracts and then exposed them to Aβ25-35. The water extracts of *H. cordata* Thunb. (Saururaceae) could inhibit the Aβ(25-35)-induced increase in the intracellular Ca^2+^ level, overproduction of ROS, disruption of the mitochondrial membrane potential, and activation of caspase 3, which blocked Aβ(25-35)-induced toxicity. Yang et al. (2012) were the first to show the concomitant beneficial effects of huperzine A [a novel alkaloid isolated from *Huperzia serrata* (Thunb.) Trevis. (Lycopodiaceae)] on mitochondrial dysfunction and memory deficits in AβPP/PS1 double transgenic mice at a time point that acetylcholinesterase (AChE) was not inhibited, suggesting that these effects were independent of its recognized inhibition of AChE. Moreover, using mitochondria isolated from the cortex, huperzine A could reduce ATP production induced by oligomeric Aβ1-42 and decreased mitochondrial swelling and the enzymatic activities of respiratory chain complexes, particularly complexes II–III and IV, which may be attributed to the prevention of oligomeric Aβ1-42 from entering the mitochondria. According to Huang et al. (2016), a peroral treatment with 200 and 400 mg/kg ethyl acetate extract from *Diospyros kaki* L.f. (Ebenaceae) can reduce the apoptosis of hippocampal neurons in rat AD models induced by Aβ, while reducing the levels of p-JNK, caspase3 and the ratio of Bax to Bcl-2. *D. kaki* L.f. (Ebenaceae) extract exerts a strong protective effect on the Aβ-induced cognitive dysfunction in rats and this effect is related to the regulation of the antioxidant defense system and mitochondrial apoptosis pathway.

### Treatments Targeting the Tau Protein

#### Inhibition of Tau Protein Hyperphosphorylation

Currently, the hyperphosphorylation of tau protein is believed to play a key role in promoting neurodegeneration in patients with AD by decreasing the biological activity of the tau protein and leading to the loss of microtubule stability. Recently, the administration of some natural drugs to AD models has been shown to inhibit the hyperphosphorylation of the tau protein by regulating the activity of cyclin-dependent kinase-5 (Gao et al., 2014; Ma et al., 2015), glycogen synthase kinase-3 (Li et al., 2011a) or serine/threonine protein phosphatase-2a (Karakani et al., 2015) to improve the symptoms of AD model rats.

##### Cdk5 Inhibitors

Feng et al. (2017) treated rat models of AD induced by Aβ1-40 and *Amanita phalloides* with Tongmai Yizhi Decoction (TYD) and huperzine A. TYD included six raw materials: *A. oxyphylla* Miq. (Zingiberaceae) (fructus, stir-heated), *R. multiflora* (Thunb.) Moldenke (Polygonaceae) (root, steamed with soya-bean milk), *C. anthriscoides* (H. Boissieu) Pimenov and Kljuykov (Apiaceae) (root and rhizome), Borneolum syntheticum (crystallization), *P. tenuifolia* Willd. (Polygalaceae) (root) and *Whitmania pigra* Whitman (Haemopidae) (entire body), all of them were mixed by 75:50:50:50:15:1. The result showed the escape latency of the model rats was significantly decreased. Compared with the model group, the pathological changes mediated by Aβ1-40 in the hippocampus and ibotenic acid (IBO) were reversed in the treatment group. HE staining revealed significantly decreased Cdk5 and Cdk5 expression in the hippocampus of model rats. Based on these results, the treatment could inhibit Cdk5 expression and reduce protein phosphorylation (**Table 1**). Ma et al. (2015) found that safflower yellow (SY) from *Carthamus tinctorius* L. (Asteraceae) could inhibit the activation of the GSK-3 and GSK-5 signaling pathways in AD model rats, thereby inhibiting the morphological changes in neurons and the hyperphosphorylation of the tau protein caused by Aβ1-42 and significantly reducing the learning and memory impairment mediated by Aβ1-42 in the hippocampus of model rats.

**Table 1:** Treatments targeting the tau protein.

Study on botanical or multiherbal	Species (Family), source, concentration			Correct species name published? (Y/N)	Chemical analysis reported? (Y/N)	Quality control reported? (Y/N)	Type of study	Mechanism of action
(Chang et al., 2016)	*Glycyrrhiza inflata* Batalin (Fabaceae), purchased from Sun Ten Pharmaceutical Co Ltd (Taipei, Taiwan, Republic of China), 100 g			N - *G. inflata*	Y - HPLC	Y - TLC	*In vitro*	Mutant tau protein repeat domain↓, nerve axon growth↑, ERN2↑, unfolded protein↑, SERP1↓
	*Panax ginseng* C.A.Mey. (Araliaceae), Purchased from Sun Ten Pharmaceutical Co Ltd (Taipei, Taiwan, Republic of China), 100 g			N - *P. ginseng*				Mutant tau protein repeat domain↓, nerve axon growth↑
Study on patented formulation	Name	Source	Species (Family), parts used, concentration	Correct species name published? (Y/N)	Chemical analysis reported? (Y/N)	Quality control reported? (Y/N)	Type of study	Mechanism of action
(Feng et al., 2017)	TYD	Purchased from Bozhou, China and prepared by (Feng et al., 2017)	*Alpinia oxyphylla* Miq. (Zingiberaceae), fructus, 75	N - *Fructus* *Alipiniae Oxyphyllae*	Y - HPLC	Y - Prepared according to the People's Republic of China (2010 edition) pharmacopeia	*In vivo*	Cdk5 expression↓
			*R. multiflora* (Thunb.) Moldenke (Polygonaceae), root, 50	N - *Radix Polygoni Multiflori Preparata*				
			*Conioselinum anthriscoides* (H.Boissieu) Pimenov & Kljuykov (Apiaceae), root and rhizome, 50	N - *Rhizoma Ligustici Chuangxiong*				
			Borneolum syntheticum, crystallization, 50	Y				
			*Polygala tenuifolia* Willd. (Polygalaceae), root, 15	N - *Radix Polygalae*				
			*Whitmania pigra* Whitman (Haemopidae), entire body, 1	Y				
Study on isolated chemical compound	Compound, molecular formula, concentration	Source	Species (Family)	Correct species name published? (Y/N)	Purity (%) reported? (Y/N)	Quality control reported? (Y/N)	Type of study	Mechanism of action
(Feng et al., 2017)	Huperzine A, C_15_H_18_N_2_O, 50 μg	Purchased from Chenxin Pharmaceutical Co. China; H20093133	*Huperzia serrata* (Thunb.) Trevis. (Lycopodiaceae)	Y	N	Y	*In vivo*	Cdk5 expression↓
(Ma et al., 2015)	SY, C_43_H_44_O_24_, 10/30/100 mg/kg	Purchased from Yunnan Tonghai Yangshi Biotechnology Co., Ltd	*Carthamus tinctorius* L. (Asteraceae)	N - C*. tinctorius*	Y - > 99%	Y	*In vivo*	GSK-3 and GSK-5 signalings↓
(Gao et al., 2014)	Geniposide, C_17_H_24_O_10_, 50 μM	Purchased from Marker Inc (Tianjin, China)	*Gardenia jasminoides* J.Ellis (Rubiaceae)	N - *G. jasminoides* Ellis	N	Y	*In vivo*	GSK3β overactivity↓
(Li et al., 2011a)	Ginsenoside Rd, C_48_H_82_O_18_, 10mg/kg	Purchased from Tai-He Biopharmaceutical Co., Ltd., Guangzhou, China	*P. ginseng* C.A.Mey. (Araliaceae)	N – *P. ginseng*	N	Y	*In vivo and vitro*	PP-2A↑
(Karakani et al., 2015)	Crocin, C_44_H_64_O_24_, 2 mg/ml	Purified by (Karakani et al., 2015)	*Crocus sativus* L. (Iridaceae)	N - C*. sativus L.*	N	Y	*In vitro*	Interfere tau nucleation phase
(Gong et al., 2014)	1,4-benzoquinone, C_6_H_4_O_2_, mixed with insulin in a molar ratio of protein: compound=2:1	Purchased from Aladdin-Reagents (Shanghai, China)	Widely exist in medicinal plants or food	Y	N	Y	*In vitro*	Amyloid fibers↓, hemolysis level↓, insulin form toxic oligomers↓
	1,4-naphthoquinon, C_10_H_6_O_2_, same concentration							
	9,10-anthraquinone, C_14_H_8_O_2_, same concentration							
	9,10-phenanthraquin one, C_14_H_8_O_2_, same concentration							
	Chrysophanol, C_15_H_10_O_4_, same concentration		*Rheum rhabarbarum* L. (Polygonaceae)					
	Emodin, C_15_H_10_O_5_, same concentration							
	Aloe-emodin, C_15_H_10_O_5_, same concentration							
	Rhein, C_15_H_8_O_6_, same concentration							
(Azimi et al., 2016)	α-cyperone, C_15_H_22_O, 1 μg	Purchased from Chem Faces (Wuhan, China)	*Cyperus rotundus* L. (Cyperaceae)	Y	N	Y	*In vitro*	Unstable tubulin aggregation↓, inflammation↓

TLC, Thin layer chromatography; TYD, Tongmai Yizhi Decoction; HPLC, high-performance liquid chromatography; Cdk5, cyclin-dependent kinase-5; SY, safflower yellow; GSK, glycogen synthase kinase; PP-2A, protein phosphatase 2; ERN, endoplasmic reticulum to nucleus signaling; SERP, stress-associated endoplasmic reticulum protein 1.

##### GSK3β Inhibitors

A streptozotocin (STZ) injection is known to increase the activity of GSK-3β and cause the hyperphosphorylation of the tau protein. Gao et al. (2014) administered geniposide [50 M, 10 L, isolated from the fruit of *G. jasminoides* J.Ellis (Rubiaceae)] through oral gavage or unilateral injections into the ventricle of AD model mice treated with STZ and found that geniposide could reduce the hyperactivity of GSK3β induced by STZ by western blotting analysis. Meanwhile, geniposide improved the spatial learning of rats. According to this new evidence, the active monomer of geniposide can be used as a new therapeutic agent for AD (Gao et al., 2014).

##### PP-2a Activator

Li et al. (2011a) microinjected okadaic acid (OA) (protein phosphatase inhibitor) into both ventricles of adult male SD rats or added it to the cortical neuron culture medium to stimulate the hyperphosphorylation of tau *in vivo* or *in vitro*. After 7 days of pretreatment with 10 mg/(kg·day) ginsenoside Rd from *Panax ginseng* C.A.Mey. (Araliaceae) in SD rats or cultured cortical neurons (2.5 or 5 mol/L, 12 h), the activity of PP-2A was increased and the neurotoxicity induced by OA and the hyperphosphorylation of tau were decreased.

#### Inhibition of Tau Protein Aggregation

Several synthetic drugs that are currently used to inhibit tau aggregation have moderate beneficial effects, but induce numerous side effects. Therefore, the development of natural drugs with anti-tau aggregation properties is anticipated. The tau protein is stable in fibrils and exhibits a low aggregation tendency in the presence of crocin. Based on transmission electron microscopy, crocin from *Crocus sativus* L. (Iridaceae) could interfere with the nucleation of the tau protein and inhibits the formation of tau protein filaments (Karakani et al., 2015).

Gong et al. (2014) used insulin as an amyloid protein model to test four simple quinones (1,4-benzoquinone, 1,4-naphthoquinone, 9,10-anthraquinone and 9,10-phenanthraquinone) from medicinal plants or food and four Anthraquinone derivatives (chrysophanol, emodin, aloe-emodin, and rhein) from *Rheum rhabarbarum* L. (Polygonaceae). After treatment with these quinones, the number of amyloid-mimicking insulin fibers decreased, and the level of hemolysis decreased. Thus, quinones can inhibit the formation of toxic insulin oligomers, and its derivatives may play a potential role in preventing protein misfolding diseases.

The extracts of *Glycyrrhiza inflata* Batalin (Fabaceae) and *P. ginseng* C.A. Mey. (Araliaceae) also have the potential to improve the growth of mutant tau protein repeat domain (K280 tauRD DsReSH-SY5Y) and axons in neuronal cell model. *G. inflata* Batalin (Fabaceae) can further upregulate Ern2 and increase the unfolded protein response by downregulating Serp1 in chaperone Dnajc3 and K280 tauRDDs Red 293 cells (Chang et al., 2016).

*In vitro*, α-cyperone from rhizomes of *Cyperus rotundus* L. (Cyperaceae) exerts a significant effect on the tubulin structure by reducing the rate of polymerization and concentration of polymerized tubulin. The interaction between α-cyperone and tubulin can reduce the aggregation of unstable tubulin, which may inhibit inflammation and represent a beneficial treatment for AD (Azimi et al., 2016).

### Relevant Therapy Based on Oxidative Stress Inhibition

#### Promotion of Free Radical Scavenging

Superoxide dismutase (SOD) and glutathione peroxidase (GSH-Px) are important enzymes that scavenge free radicals in cells. The free radical scavenging capacity is increased by increasing the activities of these enzyme. Malondialdehyde (MDA) is one of the products of lipid oxidation. Lipid oxidation can cause the cross-linking polymerization of proteins, nucleic acids and other macromolecules, and thus exerts cytotoxic effects. Therefore, the reduction of intracellular lipid oxidation and MDA production can play an antioxidant role.

The oral administration of an ethyl acetate extract of *D. kaki* L.f. (Ebenaceae) (Huang et al., 2016), oral administration of Bushen-Yizhi formula (BSYZ) (Hou et al., 2014), intragastric administration of SY (Ma et al., 2015) and inhalation of volatile oil of *C. sativum* L. (Apiaceae) (Cioanca et al., 2013) could increase SOD and GSH-Px activity and decrease MDA levels in brain tissues from AD model rats, thereby reducing oxidative stress in the brain. At the same time, the memory and cognitive impairments of model rats are significantly improved. In addition, the pathology showed that SY could inhibit the hyperphosphorylation of the tau protein and morphological changes in hippocampal neurons (Ma et al., 2015). BSYZ consisted of six medicinal plants, fruit of *Cnidium monnieri* (L.) Cusson (Apiaceae), rhizome of *P. ginseng* C.A. Mey. (Araliaceae), preparata of *R. multiflora* (Thunb.) Moldenke (Polygonaceae), cortex of *Paeonia × suffruticosa* Andrews (Paeoniaceae), fruit of *Ligustrum lucidum* W.T.Aiton (Oleaceae) and *Lycium barbarum* L. (Solanaceae) mixed in a ratio of 3:3:2:2:2:2, which can inhibit neuronal apoptosis and exert an anti-AD effect as volatile oil of *C. sativum* L. (Apiaceae) (**Table 2**). Li et al. (2014) performed the Y-maze test, shuttle box test and Morris water maze test and found that injections of 0.01 or 0.1 mg/kg schisantherin A from *S. chinensis* (Turcz.) Baill. (Schisandraceae) in the lateral ventricle for 5 consecutive days could significantly reduce learning and memory impairments induced by Aβ1-42. In addition, Mao et al. (2015) injected schisandrin C [extracted from the fruits of *S. chinensis* (Turcz.) Baill. (Schisandraceae)] into the cerebellar ventricle for 5 consecutive days. Compared with the sham operation group, Aβ1-42 levels and neuronal damage were significantly reversed in Aβ-induced AD model rats; meanwhile, short-term and working memory impairments were also substantially improved.

**Table 2:** Relevant therapy based on oxidative stress inhibition.

Study on botanical or multiherbal	Species (Family), source, concentration			Correct species name published? (Y/N)	Chemical analysis reported? (Y/N)	Quality control reported? (Y/N)	Type of study	Mechanism of action
(Huang et al., 2016)	*D. kaki* L.f. (Ebenaceae), collected in November, 2013 from Bengdu city of Anhui province in China, 3 kg			N - D. kaki Thunb.	Y - HPLC	Y - Prepared according to part IV of the Chinese Pharmacopoeia 2015 edition	*In vivo*	SOD and GSH-Px activity↑, MDA level↓
(Cioanca et al., 2013)	*Coriandrum sativum* L. (Apiaceae), collected from the experimental fields of Agricultural Research and Development Center, Secuieni, 157 Neamt (Eastern Romania)			Y	Y - GC/MS, GC/FID	Y – Prepared according to European Pharmacopoeia (Ph. Eur.) 6th edition, 2008	*In vivo*	SOD and GSH-Px activity↑, MDA level↓, neuronal apoptosis↓
(Gao et al., 2013a)	*S. sanghuang* (Sheng H. Wu, T. Hatt. & Y.C. Dai) Sheng H. Wu, L.W. Zhou & Y.C. Dai, 2015 (Sanghuangporus), purchased from Hun'chun lvdao medicinal Co. Ltd. (Hn'chun,China), 1 kg			N - Phellinus linteus	Y - HPLC	Y – Entire body ground, boiled, cooled, filtered, evaporated and freeze-dried	*In vitro*	ROS↓, ΔΨm↑, 8-OHdG↑, HepG2 cells toxicity↓
Study on patented formulation	Name of preparation	Source	Species (Family), parts used, concentration	Correct species name published? (Y/N)	Chemical analysis reported? (Y/N)	Quality control reported? (Y/N)	Type of study	Mechanism of action
(Hou et al., 2014)	BSYZ	Purchased from the National Institutes for Food and Drug Control (Beijing, China)	*Cnidium monnieri* (L.) Cusson (Apiaceae), fruit, 3	Y	Y - HPLC	Y - Prepared according to the People's Republic of China (2010 edition) pharmacopeia	*In vivo*	SOD and GSH-Px activity↑, MDA level↓, neuronal apoptosis↓
			*P. ginseng* C.A.Mey. (Araliaceae), rhizome, 3	Y				
			*R. multiflora* (Thunb.) Moldenke (Polygonaceae), radix, 2	N - *Polygonum multiflorum* Thuna.				
			*Paeonia × suffruticosa* Andrews (Paeoniaceae), cortex, 2	Y				
			*Ligustrum lucidum* W.T.Aiton (Oleaceae), fruit, 2	Y				
			*Lycium barbarum* L. (Solanaceae), fruit, 2	Y				
(Shu et al., 2017)	Zishenhuoxuefang	Purchased from ARK PHA.LTD (Shanxi, China)	*Rehmannia glutinosa* (Gaertn.) DC. (Orobanchaceae), 30 g	Y	Y - HPLC	Y - Prepared according to the People's Republic of China (2010 edition) pharmacopeia	*In clinic (II)*	SOD↑, GSH-Px↑
			*R. multiflora* (Thunb.) Moldenke (Polygonaceae), 20 g	Y				
			*C. anthriscoides* (H.Boissieu) Pimenov & Kljuykov (Apiaceae), 15 g	Y				
			*L. barbarum* L. (Solanaceae), 15 g	Y				
			*C. monnieri* (L.) Cusson (Apiaceae), 15 g	Y				
			*P. ginseng* C.A.Mey. (Araliaceae), 15 g	Y				
			*Salvia miltiorrhiza* Bunge (Lamiaceae), 15 g	Y				
			Aulastomum gulo, 10 g	Y				
			*L. lucidum* W.T.Aiton (Oleaceae), 10 g	Y				
			*Acorus calamus* var. *angustatus* Besser (Acoraceae), 10 g	Y				
			*Polygala sibirica* L. (Polygalaceae), 10 g	Y				
			*Glycyrrhiza uralensis* Fisch. ex DC. (Fabaceae), 10 g	Y				
Study on isolated chemical compound	Compound, molecular formula, concentration	Source	Species (Family)	Correct species name published? (Y/N)	Purity (%) reported? (Y/N)	Quality control reported? (Y/N)	Type of study	Mechanism of action
(Ma et al., 2015)	SY, C_43_H_44_O_24_, 10/30/100 mg/kg	Provided by Yunnan Tonghai Yangshi Biotechnology Co., Ltd	*C. tinctorius* L. (Asteraceae)	N - C*. tinctorius*	Y - > 99%	Y	*In vivo*	SOD and GSH-Px activity↑, MDA level↓, tau protein hyperphosphorylation↓, neuronal morphological changes↓
(Li et al., 2014)	Schisantherin A, C_30_H_32_O_9_, 0.01/0.1 mg/kg	Provided by the National Institute for the Control of Pharmaceutical and Biological Products in China (Shenyang, China)	*Schisandra chinensis* (Turcz.) Baill. (Schisandraceae)	Y	Y – > 98%	Y	*In vivo*	Oxidative stress↓
(Mao et al., 2015)	Schisandrin C, C_22_H_24_O_6_, 0.2/2 μg/μL	Provided by Weikeqi Institute of Biotechnology (Sichuan, China)	*S. chinensis* (Turcz.) Baill. (Schisandraceae)	Y	Y - > 98%	Y	*In vivo and vitro*	Oxidative stress↓
(Singh et al., 2015)	Vanillic acid, C_8_H_8_O_4_, 25/50/100 mg/kg	Purchased from Sigma-Aldrich, St. Louis, MO.	*Angelica sinensis* (Oliv.) Diels (Apiaceae)	Y	N	Y	*In vivo*	AChE↓, corticosterone↓, TNF-α↓, oxidative stress↓
(Ajith and Padmajanair, 2015)	Coenzyme Q10, C_59_H_90_O_4_	Reviewed by (Ajith and Padmajanair, 2015)	Widely exist in medicinal plants or food	Y	N	Y	*In vivo and vitro*	Mitochondrial function↑, oxidative stress↓
	α-lipoic acid, C_8_H_14_O_2_S_2_			Y	N	Y		
	Selenium, Se			Y	N	Y		
	ω-3 fatty acid, C_60_H_92_O_6_			Y	N	Y		
	Vitamin E, C_29_H_50_O_2_			Y	N	Y		
(Lee et al., 2015)	Acerogenin A, C_19_H_22_O_3_, 30 μM	Provided from Prof. Byung-Yoon Cha	*Parthenocissus tricuspidata* (Siebold & Zucc.) Planch. (Vitaceae)	N - *Acer nikoense* Maxim (Aceraceae)	N	N	*In vitro*	PI3K/Akt and Nrf2 pathway, HO-1 regulation
(Lee et al., 2016)	DTMF, C_18_H_16_O_7_, 1/5/10/20/40 μM	Purchased from the Kyungdong traditional herbal market (Seoul, Korea)	*Siegesbeckia pubescens* (Makino) Makino (Compositae)	N -*S. pubescens*	N	Y	*In vivo and vitro*	iNOS↓,COX-2↓, HO-1 expression, HO activity, Nrf2 nuclear translocation, ARE-luciferase activity
(Suganthy et al., 2016)	Different kinds of quercetin and derivatives	Reviewed by (Suganthy et al., 2016)	Widely exist in medicinal plants or food (USDA, 2013; quercetin.com)	Y	Y – See USDA, 2013; quercetin.com	Y	*In vivo and vitro*	Nrf2-dependent antioxidant response elements, STAT-1↓, kinase signaling cascades
(Zhang et al., 2016a)	Gas, C_13_H_18_O_7_, 60 mg/kg	Purchased from Sigma–Aldrich, 108 Louis, MO, USA	*Gastrodia elata* Blume (Orchidaceae)	N - *G. elata* Blume	N	Y	*In vivo and vitro*	SOD, CAT, MDA and ROS↓, PKR/eIF2α↓

SOD, superoxide dismutase; GSH-Px, glutathione peroxidase; MDA, malondialdehyde; BSYZ, Bushen-Yizhi formula; AChE, acetylcholinesterase; TNF, tumor necrosis factor; ROS, reactive oxygen species; ΔΨm, membrane potential; 8-OHdG, 8-hydroxydeoxyguanosine; HepG2, liver hepatocellular carcinoma; PI3K, phosphoinositide 3-kinase; Akt, protein kinase B; Nrf2, nuclear factor-E2-related factor; HO, heme oxygenase; DTMF, 5,3′-Dihydroxy-3,7,4-trimethoxyflavone; iNOS, inducible nitric oxide synthase; COX-2, cyclooxygenase; ARE, antioxidant response element; STAT-1, signal transducer and activator of transcription-1; Gas, gastrodin; CAT, catalase; MDA, malondialdehyde; PKR/eIF2α, protein kinase *R*/eukaryotic initiation factor-2α.

Singh et al. (2015) established and compared five groups of mice, including a negative control and three groups treated with 25, 50 or 100 mg/kg vanillic acid from *A. sinensis* (Oliv.) Diels (Apiaceae) for 28 days. At doses of 50 and 100 mg/kg, vanillic acid significantly improved memory and decreased AChE and corticosterone TNF-α levels. In addition, 100 mg/kg vanillic acid exerted a dose-dependent effect on some parameters. Vanillic acid could improve spatial learning and memory retention by preventing oxidative stress.

In a clinical trial, 120 patients with AD were randomly divided into two groups. The control group was orally administered donepezil hydrochloride and piracetam. The observation group was administered 60 Zishenhuoxuefang's synergism treatments and compared with the control group. Zishenhuoxuefang consist of *R. glutinosa* (Gaertn.) DC. (Orobanchaceae) (30 g), *R. multiflora* (Thunb.) Moldenke (Polygonaceae) (20 g), *C. anthriscoides* (H.Boissieu) Pimenov and Kljuykov (Apiaceae) (15 g), *L. barbarum* L. (Solanaceae) (15 g), *C. monnieri* (L.) Cusson (Apiaceae) (15 g), *P. ginseng* C.A.Mey. (Araliaceae) (15 g), *S. miltiorrhiza* Bunge (Lamiaceae) (15 g), *Aulastomum gulo* (10 g), *L. lucidum* W.T.Aiton (Oleaceae) (10 g), *Acorus calamus* var. *angustatus* Besser (Acoraceae) (10 g), *Polygala sibirica* L. (Polygalaceae) (10 g), *Glycyrrhiza uralensis* Fisch. ex DC. (Fabaceae) (10 g). After treatment, the levels and activity of SOD and GSH-Px increased, and the level of Aβ decreased in the two groups, and the observation group recorded better scores than the control group (Shu et al., 2017).

#### Inhibition of Free Radicals Production

Excessive production of ROS leads to oxidative stress, which is closely related to the development of AD. Mitochondria are the main site of ROS production in cells, and are vulnerable to oxidative stress. Oxidative stress attacks the mitochondrial DNA, resulting in abnormal electron transport chains and energy production barriers (Ajith and Padmajanair, 2015). Currently, mitochondrial dysfunction is acknowledged to be associated with neurodegenerative diseases, and mitochondrial defects are even regarded as the core factor of AD progression (Cardoso et al., 2017). Serious deficiencies in energy metabolism are observed during the early stage of AD, which may be due to the decrease in activity of complexes in the electron transport chain (ETC) and damage to the mitochondrial DNA induced by Aβ and ROS. The antioxidant coenzyme Q10, α-lipoic acid, selenium, ω-3 fatty acid and vitamin E improve mitochondrial function and reduce oxidative stress. These compounds are accepted as adjuvant drugs for mild to moderate dementia (Ajith and Padmajanair, 2015). *Sanghuangporus sanghuang* (Sheng H. Wu, T. Hatt. & Y.C. Dai) Sheng H. Wu, L.W. Zhou & Y.C. Dai, 2015 (Sanghuangporus) reduces ROS formation in HepG2 (liver hepatocellular carcinoma) cells. Based on a recent validation study, *S. sanghuang* (Sheng H. Wu, T. Hatt. & Y.C. Dai) Sheng H. Wu, L.W. Zhou & Y.C. Dai, 2015 (Sanghuangporus) significantly reduced the ROS production induced by tacrine, the damage to the ΔΨm (membrane potential), the damage to the mitochondrial DNA caused by 8-hydroxydeoxyguanosine (8-OHdG) formation, and the toxicity in HepG2 cells to prevent mitochondrial damage (Gao et al., 2013a). As the number of scientific investigations increases, “mitochondrial medicine” has been proposed and has become a new therapeutic strategy to maintain energy production and inhibit neuronal apoptosis.

#### Regulation of Oxidative Stress Pathway

Heme oxygenase-1 (HO-1) plays a key role in the pathogenesis of neuronal diseases and oxidative stress. According to Lee et al. (2015), the natural compound acerogenin A from *Parthenocissus tricuspidata* (Siebold and Zucc.) Planch. (Vitaceae) could effectively prevent glutamate-induced oxidative damage in HT22 cells by regulating HO-1 expression through the phosphoinositide 3-kinase/protein kinase B (PI3K/Akt) and nuclear factor-E_2_-related factor (Nrf2) pathway; this compound exerted a neuroprotective effect.

5,3′-Dihydroxy-3,7,4-trimethoxyflavone (DTMF) from *Siegesbeckia pubescens* (Makino) Makino (Compositae) can reduce the LPS-induced expression of inducible nitric oxide synthase (iNOS) and cyclooxygenase (COX-2) and promote HO-1 expression, HO activity, Nrf2 nuclear translocation and antioxidant response element (ARE)-luciferase activity, suggesting that it may represent an effective treatment for neurodegenerative diseases caused by oxidative stress (Lee et al., 2016). As shown in the study by Suganthy et al. (2016), quercetin alleviates nerve injury induced by oxidative stress by regulating the expression of Nrf2-dependent antioxidant response elements and inhibiting NF-κB and signal transducer and activator of transcription-1 (STAT-1) signaling to alleviate neuroinflammation by regulating a large number of kinase signaling cascades, such as tyrosine kinase C and protein kinase C (PKC), nerve repair can be enhanced and neuronal survival can be prolonged. Quercetin is widely distributed in plants such as *Allium cepa* L. (Amaryllidaceae), *Asparagus officinalis* L. (Asparagaceae), and *Lactuca sativa* L. (Asteraceae) etc. Zhang et al. (2016a) measured SOD activity, catalase (CAT) activity, MDA activity and ROS levels to assess the oxidative stress level in the brains of Tg2576 mice. The glucoside gastrodin (Gas) from *Gastrodia elata* Blume (Orchidaceae) could reduce oxidative stress in the hippocampus of Tg2576 transgenic mice and improves learning and memory. In addition, Gas decreases β-secretase expression by inhibiting the activity of protein kinase R/eukaryotic initiation factor-2α (PKR/eIF2α) in SH-SY5Y cells treated with hydrogen oxide.

### Relevant Therapies for Inhibiting Neuroinflammatory Factors

#### Regulation of Inflammatory Cytokines

Many inflammatory factors are involved in the pathogenesis of AD. Tumor necrosis factor (TNF-α), interleukin-1β (IL-1β) and interleukin-6 (IL-6) play prominent roles. Increased TNF-α levels in the cerebrospinal fluid of patients with AD can lead to synaptic dysfunction and amyloid production, resulting in memory impairment.

The expression of TNF-α, IL-1β and IL-6 in the hippocampus of a mouse model of AD induced by Aβ were decrease after treatment with TianDiJingWan (TDJW) (Li et al., 2015c), Compound Danshen Tablets (CDT) (Teng et al., 2014) and the combination of ginkgo flavonoids from *Ginkgo bilob*a L. (Ginkgoaceae) and polysaccharides from *Polystictus versicolor* (L.) Fr. (Polyporaceae) (Fang et al., 2015) (**Table 3**). Also, the memory impairment was improved. TDJW is consists of six kinds of TCM including *Pheretima aspergillum* E. (Geosaurus) (3 g), *W. pigra* Whitman (Haemopidae) (1 g), rhizome of *A. calamus* var. *angustatus* Besser (Acoraceae) (6 g), tuber of *G. elata* Blume (Orchidaceae) (10 g), rhizome of *Polygonatum sibiricum* Redouté (Asparagaceae) (10 g), and fruit of *L. lucidum* W.T.Aiton (Oleaceae) (10 g), which decreased Aβ and p-tau levels and increased acetylcholine and glutamic acid levels in the CA1 region of hippocampus in AD mice. CDT is consisted of *S. miltiorrhiza* Bunge (Lamiaceae), *P. notoginseng* (Burkill) F.H.Chen (Araliaceae), borneol in proportions of 450:141:8, which increased the level of brain-derived neurotrophic factor (BDNF), while the combination of ginkgo flavonoids and polysaccharides could increase SOD levels and, subsequently, decrease the intracerebral oxidation level. The ayurvedic plant *Bacopa monnieri* (L.) Wettst. (Plantaginaceae) can inhibit not only the production of the inflammatory cytokine TNF-α but also the release of IL-6 and the activation of cerebral inflammation-associated enzymes (caspase-3 or -5) in cultured microglia *in vitro* to exert an anti-inflammatory effect on the central nervous system (Nemetchek et al., 2017). In a clinical trial, Li et al. (2015a) divided participants with moderate AD into three groups, a group treated with *Cistanche deserticola* Ma (Orobanchaceae) (n=10), a group treated with donepezil hydrochloride tablets (n=8), and the untreated control group (n=6), to evaluate the neuroprotective effect on patients with AD. After 48 weeks of treatment, decreases in T-tau, TNF-α and IL-1β levels were observed in the *Cistanche deserticola* Ma (Orobanchaceae) group compared with the control group, indicating its neuroprotective potential.

**Table 3:** Relevant therapies for inhibiting neuroinflammatory factors.

Study on botanical or multiherbal	Species (Family), source, concentration			Correct species name published? (Y/N)	Chemical analysis reported? (Y/N)	Quality control reported? (Y/N)	Type of study	Mechanism of action
(Nemetchek et al., 2017)	*Bacopa monnieri* (L.) Wettst. (Plantaginaceae), obtained from Banyan Botanicals (Albuquerque, NM; product lot number 641,615, batch number BM468P), 10 g			Y	Y - HPTLC	Y - A phytochemical reference standard of bacopa from ChromaDex (Irvine, CA, USA)	*In vitro*	TNF-α, IL-6 and cerebral inflammatory-associated enzymes↓
(Li et al., 2015a)	*Cistanche deserticola* Ma (Orobanchaceae), LYYLYD pharmaceuticals Company, Changchun, China, 0.3g/capsule			Y	Y	Y	*In clinic (Ι)*	T-tau, TNF-α and IL-1β↓
Study on patented formulation	Name of preparation	Source	Species (Family), parts used, concentration	Correct species name published? (Y/N)	Chemical analysis reported? (Y/N)	Quality control reported? (Y/N)	Type of study	Mechanism of action
(Li et al., 2015c)	TDJW	Prepared by (Li et al., 2015c)	*Pheretima aspergillum* E. (Geosaurus), entire body, 3 g	Y	Y - HPLC	Y - Prepared according to Chinese pharmacopoeia (2010 edition)	*In vivo*	TNF-α, IL-1β and IL-6↓, Aβ and p-tau↓, ACh and glutamin acid↑
			*W. pigra* Whitman (Haemopidae), entire body, 1 g	Y				
			*A. calamus* var. *angustatus* Besser (Acoraceae), rhizome, 6 g	N - *Acorus tatarinowii* Schott				
			*G. elata* Blume (Orchidaceae), tuber, 10 g	Y				
			*Polygonatum sibiricum* Redouté (Asparagaceae), rhizome, 10 g	Y				
			*L. lucidum* W.T.Aiton (Oleaceae), fruit, 10 g	Y				
(Teng et al., 2014)	CDT	Produced by Hutchison Whampoa Guangzhou Baiyunshan Chinese Medicine Co, Ltd (batch number: Z44023372, Guangzhou, China)	*S. miltiorrhiza* Bunge (Lamiaceae), 450	N - *S. miltiorrhiza*	Y - HPLC	Y	*In vivo*	TNF-α, IL-1β and IL-6↓, BDNF↑
			*Panax Notoginseng* (Burkill) F.H.Chen (Araliaceae), 141	N - P*. notoginseng*				
			Borneol, 8	N - *Borneol*				
(Wang et al., 2014)	Qifu-Yin	Purchased from Integrated Chinese and Western Medicine Hospital of Jiangsu (Nanjing, China)	*P. ginseng* C.A.Mey. (Araliaceae), rhizoma, 6	N - Ginseng Radix et Rhizoma	N	Y - Prepared according to "Jingyue Quanshu" (a medical classic written by Jingyue Zhang in the Ming Dynasty)	*In vivo*	RAGE, NF-κB and Aβ↓, TNF-α and IL-1β↓
			*R. glutinosa* (Gaertn.) DC. (Orobanchaceae), radix, 9	N - Rehmanniae Radix Praeparata				
			*A. sinensis* (Oliv.) Diels (Apiaceae), radix, 9	N - Angelicae Sinensis Radix				
			*Atractylodes macrocephala* Koidz. (Asteraceae), rhizome, 5	N - Atractylodis Macrocephalae Rhizoma				
			*Ziziphus jujuba* Mill. (Rhamnaceae), semen, 6	N - Ziziphi Spinosae Semen				
			*P. tenuifolia* Willd. (Polygalaceae), radix, 5	N - Polygalae Radix				
			*G. inflata* Batalin (Fabaceae), rhizome, 3	N - Glycyrrhiza Radix et Rhizoma				
Study on isolated chemical compound	Compound, molecular formula, concentration	Source	Species (Family)	Correct species name published? (Y/N)	Purity (%) reported? (Y/N)	Quality control reported? (Y/N)	Type of study	Mechanism of action
(Fang et al., 2015)	Ginkgo flavonoid, not reported, 6/75/90/37.5/45 mg/kg/day	Purchased from Xian Reain Biotechnology Co., Ltd. (Xian, China)	*Ginkgo biloba* L. (Ginkgoaceae)	N - Ginkgo flavonoid	N	Y	*In vivo*	SOD↑, TNF-α, IL-1β and IL-6↓
	Polysaccharides, not reported, 6/75/90/37.5/45 mg/kg/day		*Polystictus versicolor* (L.) Fr. (Polyporaceae)	N - *Coriolus versicolor*	N			
(Yu et al., 2012)	ASD, C_47_H_76_O_18_, 30/90/270 mg/kg	Prepared by Professor Zhong-Lin Yang	*Dipsacus inermis* Wall. (Caprifoliaceae)	N - *Dipsacus* *asperoides Wall*	N	N	*In vivo*	PKB and IKK↓, NF-κB activation↓, TNF-α, IL-1β and COX-2↓
(Chen et al., 2005)	Quercetin, C_15_H_10_O_7_, 30 μM	Synthesized as (Hou et al., 2003) described	Widely exist in medicinal plants or diet	Y	Y – 99%	Y - HPLC	*In vitro*	NRF-2-dependent antioxidant elements regulation, NF-κB and STAT-1↓

TDJW, TianDiJingWan; CDT, compound Danshen tablet; IL, interleukin; ACh, acetylcholine; BDNF, brain-derived neurotrophic factor; ASD, Akebia saponin D; PKB, protein kinase B; IKK, inhibitor of nuclear factor kappa-B kinase; NF-κB, nuclear factor-kappa B; COX-2, cyclooxygenase; RAGE, advanced glycation end product receptor; NRF2, nuclear factor-E2-related factor.

#### Regulation of NF-κB Signaling Pathway

NF-κB is a nuclear transcription factor that was originally identified as binding to κB binding sites in the promoter region of the κ light chain of immunoglobin, which is present in most plasma samples. The NF-κB signal transduction pathway is activated by stimulation after injury, and the level of NF-κB is increased in many kinds of cells to mediate the inflammatory cell response and increase the expression of proinflammatory factors in the injured area, further exacerbating cerebral neuron damage (Gao et al., 2016).

*Akebia saponin* D (ASD) is a bioactive triterpenoid saponin extracted from *Dipsacus inermis* Wall. (Caprifoliaceae). After the intragastric administration of ASD to AD model mice for 4 weeks, Yu et al. (2012) observed the inhibition of both the secretion of protein kinase B (PKB) and the inhibitor of nuclear factor kappa-B kinase (IKK) in the brains of AD model mice. Meanwhile, the activation of NF-κB induced by Aβ1-42 was decreased, the activation of glia and the expression of TNF-α, IL-1β and COX-2 were decreased, whereas the patients' cognitive impairments improved.

Wang et al. (2014) administered Qifu-Yin (8.6–4.3 g/kg dayling for 30 days) to the mouse model of advanced glycation end product (AGE) induced AD, Donepezil (2 mg/kg) was administered as a positive control, and AGE + anti-advanced glycation end product receptor (anti-RAGE) were administered as an additional positive control group. Qifu-Yin is composed of seven raw materials, *P. ginseng* C.A.Mey. (Araliaceae), *R. glutinosa* (Gaertn.) DC. (Orobanchaceae), *A. sinensis* (Oliv.) Diels (Apiaceae), *Atractylodes macrocephala* Koidz. (Asteraceae), *Ziziphus jujuba* Mill. (Rhamnaceae), *P. tenuifolia* Willd. (Polygalaceae) and *G. inflata* Batalin (Fabaceae) mixed by 6:9:9:5:6:5:3, which could significantly ameliorate the AGE-induced memory impairment in a dose-dependent manner. The production of RAGE and NF-κB as well as the level of the Aβ protein in the hippocampus were substantially reduced, and TNF-α and IL-1β levels were decreased, indicating that Qifu-Yin may exert its neuroprotective effect through both the RAGE/NF-κB pathway and its anti-inflammatory activity (Wang et al., 2014). In addition, the activity of quercetin in scavenging free radicals has been confirmed. Quercetin belong to flavonol-type flavonoid, which are widely present in the diet and play a wide range of roles. By regulating the expression of NRF-2-dependent antioxidant elements, quercetin can protect neurons from oxidative stress. Neuroinflammation is also alleviated by inhibiting NF-κB signal transduction and STAT-1 (Chen et al., 2005).

### Treatments Targeting Cholinergic Neurons and Acetylcholine

Cholinergic neurons are widely distributed in the central nervous system. Cholinergic neuron injury plays an important role in AD. Changes in the levels of cholinergic markers in the basal forebrain correlate the severity of AD-related dementia in pathology studies. The activities of AChE and choline acetyltransferase (ChAT) are decreased in the brains of patients with AD, and a noticeable deficiency in ACh is observed. As a result of the decrease in cholinergic activity, ACh and the injury of various types of cholinergic receptors, multiple clinical manifestations may develop, predominantly memory and cognitive impairments (Mahlke et al., 2012; Asaduzzaman et al., 2014).

#### AChEIs

AChE is the most effective therapeutic target for the treatment of AD (Singh et al., 2013). AChEIs are the most widely used drugs to treat the symptoms of patients with mild to severe Alzheimer's disease (Geromichalos et al., 2012; Babiloni et al., 2013). Cessation may have a negative effect on cognitive and neuropsychiatric symptoms in patients who have taken AChEIs (O'Regan et al., 2015). Based on accumulating evidence, central CACE-IS targeting the blood-brain barrier are associated with a decrease in the rate of cognitive decline (Gao et al., 2013b). The available AChEIs are only effective in 20–30% of patients with AD, and at least two of the four AChEIs currently used are in fact based on ethnopharmacological research. So the development of new anti-AChE drugs from medicinal plants has attracted increasing attention (Russo et al., 2013).

Some scholars (Kaufmann et al., 2016) prepared natural water-containing crude extracts from 80 traditional Chinese medicinal plants and tested their anti-AChE activity *in vitro* using Hermann's colorimetric method. *Berberis bealei* Fortune (Berberidaceae), *Coptis chinensis* Franch. (Ranunculaceae) and *Phellodendron chinensis* C.K.Schneid. (Rutaceae) contained large amounts of isoquinoline alkaloids, which could effectively inhibit AChE activity. Combinations of individual alkaloids, such as berberine, coptisine and palmatine, synergistically enhance the inhibitory effect on ACh. Based on these results, some Chinese herbs exert synergistic inhibitory effects on AChE through their secondary metabolites.

Ohba et al. (2015) repeatedly administered a *H. serrata* (Thunb.) Trevis. (Lycopodiaceae) extract to AD model mice. This extract inhibits AChE activity while ameliorating the cognitive impairment of the mice with AD, suggesting broad application prospects of this extract as a treatment for AD symptoms. A team of researchers subsequently isolated highly effective components from *H. serrata* (Thunb.) Trevis. (Lycopodiaceae) and related plants and synthesized complete lycopodium alkaloids with a special skeleton from a genetic and biological perspective (Yang et al., 2016) (**Table 4**).

**Table 4:** Treatments targeting cholinergic neurons and acetylcholine.

Study on botanical or multiherbal	Species (Family), source, concentration			Correct species name published? (Y/N)	Chemical analysis reported? (Y/N)	Quality control reported? (Y/N)	Type of study	Mechanism of action
(Huang et al., 2013)	*G. elata* Blume (Orchidaceae), provided by Muju Tianma Native Local Industrial Center, Korea, 500 or 1 000 mg/2 mL per kg body weight			Y	N	Y - Prepared according to Chinese pharmacopeia	*In vivo*	Amyloid desposition↓, ChAT↑
(Li et al., 2016c)	*Xanthoceras sorbifolium* Bunge (Sapindaceae), collected in Chifeng city, Inner Mongolia on October 2011, 2.5/5/10 mg/kg			N - Xanthoceras sorbifolia	N	Y - Prepared according to Chinese Food and Drug Administration, Approval number: Z20040007, 2004	*In vivo*	PSD95 protein↑, BDNF↑, RhoA↓, ROCK2↓, improve the density of dendritic spine
Study on patented formulation	Name of preparation	Source	Species (Family), parts used, concentration	Correct species name published? (Y/N)	Chemical analysis reported? (Y/N)	Quality control reported? (Y/N)	Type of study	Mechanism of action
(Teng et al., 2014)	CDT	Produced by Hutchison Whampoa Guangzhou Baiyunshan Chinese Medicine Co, Ltd (batch number: Z44023372, Guangzhou, China)	*S. miltiorrhiza* Bunge (Lamiaceae), 450	N - *S. miltiorrhiza*	Y - HPLC	Y	*In vivo*	ChAT↑, BDNF↑, PKC receptor↑
			*P. notoginseng* (Burkill) F.H.Chen (Araliaceae), 141	N - P*. notoginseng*				
			Borneol, 8	N - *Borneol*				
(Hou et al., 2015)	BSYZ	Purchased from the National Institutes for Food and Drug Control (Beijing, China)	*C. monnieri* (L.) Cusson (Apiaceae), fruit, 3	Y	Y - HPLC	Y - Prepared according to the People's Republic of China (2010 edition) pharmacopeia	*In vivo*	NGF↑, TrkA↑, p75mRNA↑, ChAT↑
			*P. ginseng* C.A.Mey. (Araliaceae), rhizome, 3	Y				
			*R. multiflora* (Thunb.) Moldenke (Polygonaceae), radix, 2	N - *Polygonum multiflorum* Thuna.				
			*Paeonia × suffruticosa* Andrews (Paeoniaceae), cortex, 2	Y				
			*L. lucidum* W.T.Aiton (Oleaceae), fruit, 2	Y				
			*L. barbarum* L. (Solanaceae), fruit, 2	Y				
(Park et al., 2016)	PMC-12	Obtained from Hwalim Natural Drugs (Busan, Korea)	*R. multiflora* (Thunb.) Moldenke (Polygonaceae), root, 25.5 kg	N - *Polygonum multiflorum*	N	Y	*In vivo*	BDNF↑, synaptic level↑, hippocampal neurogenesis↑
			*P. tenuifolia* Willd. (Polygalaceae), root, 7.5 kg	N - P*. tenuifolia*				
			*R. glutinosa* (Gaertn.) DC. (Orobanchaceae), root, 9.5 kg	N - *R. glutinosa*				
			*Acorus gramineus* Aiton (Acoraceae), root, 7.5 kg	N - *Acorus gramineus*				
Study on isolated chemical compound	Compound, molecular formula, concentration	Source	Species (Family)	Correct species name published? (Y/N)	Purity (%) reported? (Y/N)	Quality control reported? (Y/N)	Type of study	Mechanism of action
(Ohba et al., 2015)	Huperzine A, C_15_H_18_N_2_O, 1 mg/mL	Collected from Yamagata city in the Gifu Prefecture	*H. serrata* (Thunb.) Trevis. (Lycopodiaceae)	N - *H. serrata*	Y - > 97%	Y - HPLC	*In vivo*	AChE↓
(Kaufmann et al., 2016)	Berberine, C_20_H_18_NO_4_^+^, 50 mg/mL	Provided by Prof. Thomas Efferth, Johannes Gutenberg University Mainz, Germany and Fluka/Sigma-Aldrich (Steinheim, Germany)	*Berberis bealei* Fortune (Berberidaceae)	N - *B. bealei*	N	Y – HPLC and MS	*In vitro*	AChE↓
	Coptisine, C_19_H_14_NO_4_^+^, 50 mg/mL		*Coptis chinensis* Franch. (Ranunculaceae)	N - C*. chinensis*				
	Palmatine, C_21_H_22_NO_4_^+^, 50 mg/mL		*Phellodendron chinensis* C.K.Schneid. (Rutaceae)	N - P*. chinensis*				
(Geromichalos et al., 2012)	Crocetin, C_20_H_24_O_4_, 0−8 mM/0−256 μM	Provided by the Cooperative Association of Krokos in Kozani, in West Macedonia, Greece	*C. sativus* L. (Iridaceae)	N - Saffron	Y - > 98%	Y - HPLC	*In vitro*	AChE↓
			Dimethylcrocetin, C_22_H_28_O_4_, 0−8 mM/0−256 μM	Y - > 98%				
	Safranal, C_10_H_14_O, 0−8 mM/0−120 μM		Purchased from Sigma-Aldrich Corp., St. Louis, MO, USA	Y - > 88%				
(Moon et al., 2014)	6-shogaol, C_17_H_24_O_3_, 5/10/20 mg/kg	Purchased from Wako Pure Chemical (Osaka, Japan)	*Zingiber officinale* Roscoe (Zingiberaceae)	N - *Z. officinale*	N	Y	*In vivo*	NGF↑, pre-/post-synaptic marker↑
(Li et al., 2016a)	TIIA, C_19_H_18_O_3_, 16/25/50/100 mg/kg	Purchased from Santa Cruz, USA	*S. miltiorrhiza* Bunge (Lamiaceae)	Y	N	Y	*In vivo*	CTF↓, BDNF↑

ChAT, choline acetyltransferase; BDNF, brain-derived neurotrophic factor; NGF, nerve growth factor; TrkA, tropomyosin receptor kinase A; PSD95, postsynaptic density protein 95; ROCK2, rho associated coiled-coil containing protein kinase 2; CTF, carboxyl terminal fragment; PMC-12, polygonum multiflorum Thunberg complex composition-12.

*C. sativus* L. (Iridaceae) extracts showed moderate AChE inhibitory activity (30%). The IC50 values of crocetin, dimethylcrocetin and safranal were 96.33 µM, 107.1 µM and 21.09 µM, respectively. Safranal only acts at the binding sites of AChE, whereas crocin and dimethyl lycopene simultaneously target binding and peripheral anion sites in the catalytic cleft. The development of new therapeutic agents based on carotenoid dual binding inhibitors will have great value (Geromichalos et al., 2012).

#### Promotion of ChAT Expression

ChAT is the main rate-limiting enzyme involved in ACh synthesis. ChAT levels are decreased in the brains of patients with AD in a manner proportional to the content of Aβ. Huang et al. (2013) intragastrically administered 500 or 1,000 mg/kg *G. elata* Blume (Orchidaceae) to a rat model of AD induced by Aβ25-35 daily for 52 days. The AD model rats that received *G. elata* Blume (Orchidaceae) exhibited significantly improved spatial memory in the Morris water maze test. Congo red staining showed a substantial decrease in the amount of amyloid deposited in the hippocampus, and ChAT expression was substantially increased in medial septum and hippocampus, as evidenced by western blot analysis. The long-term administration of *G. elata* Blume (Orchidaceae) has therapeutic potential for AD.

In a study of the mouse model of AD induced by Aβ25-35 performed by Teng et al. (2014), CDT could increase the expression of ChAT in the brain, induce BDNF production and activate the PKC receptor. The spatial memory of model mice was substantially improved in the Morris water maze test.

#### Protection of Cholinergic Neurons

The memory and cognitive impairments in patients with AD are closely related to the loss of cholinergic neurons. Nerve growth factor (NGF) can promote the regeneration of cholinergic neurons in the basal forebrain, and thus targeted transport of NGF has become a potential treatment for AD (Eyjolfsdottir et al., 2016). A variety of traditional Chinese medicine extracts exert a good therapeutic effect on AD, some of which affect the expression of NGF, BDNF and their related receptors (Hou et al., 2015).

After administering the BSYZ to the IBO-induced AD model rats, immunohistochemical staining of the brain revealed an increasing trend in the expression of NGF, TrkA and p75 in hippocampus and cortex, while ChAT and NGF expression were substantially increased. The BSYZ may improve the memory impairment of the IBO-induced AD rats by regulating NGF signal transduction and the anti-apoptotic cholinergic pathway (Hou et al., 2015). According to Moon et al. (2014), 6-shogaol, a bioactive components of Zingiber officinale Roscoe (Zingiberaceae), could increase the levels of NGF and pre-/post-synaptic markers in the hippocampus, and improves the memory impairment induced by Aβ and scopolamine.

Li et al. (2016c) treated the animal model of Aβ25-35_-_induced AD with *Xanthoceras sorbifolium* Bunge (Sapindaceae) extracts. The treatment upregulated postsynaptic density protein 95 (PSD95), the main scaffold protein in hippocampal CA1 pyramidal neurons, ameliorated the density of dendritic spines, increased the level of BDNF and decrease the expression of RhoA and its downstream target protein rho associated coiled-coil containing protein kinase 2 (ROCK2). Thus, the *X. sorbifolium* Bunge (Sapindaceae) extracts can protect dendritic spines through the BDNF signal transduction pathway and improve cognition. As shown in the study by Li et al. (2016a), TIIA could reduce the accumulation of the 1–42 CTF of the β-amyloid protein in an AD model, promote depolarization-induced BDNF synthesis without affecting total BDNF levels, and relieving the memory impairment in the AD mice, which further confirmed the protective effect of BDNF on cholinergic neurons. Moreover, Park HR et al. (Park et al., 2016) orally administered 100 or 500 mg/(kg·day) *Polygonum multiflorum* Thunberg complex composition-12 (PMC-12) to 5-week-old male C57BL/6 mice. PMC-12 is a mixture of four medicinal herbs, *R. multiflora* (Thunb.) Moldenke (Polygonaceae) (25.5 kg), *P. tenuifolia* Willd. (Polygalaceae) (7.5 kg), *R. glutinosa* (Gaertn.) DC. (Orobanchaceae) (9.5 kg), and *Acorus gramineus* Aiton (Acoraceae) (7.5 kg). After 2 weeks, the level of BDNF and number of synapses increased in the hippocampus of model mice, the neural precursor cells of dentate gyrus nerves proliferated and the number of surviving newborn cells increased. Thus, PMC-12 can promote hippocampal neurogenesis and improve neurocognitive function.

### Summary and Prospects

AD is a complex, inherited, slowly developing and irreversible neurological disease. During decades of asymptomatic progression, multiple interacting systems and molecular mechanisms contribute to the development of the early preclinical stage with situational memory impairment, as well as decline of memory and loss of cognitive function in the dementia phase. At present, it is generally believed that Aβ and Tau proteins are the main causes of AD. However, a large number of studies indicate that oxidative stress, neuroinflammatory factors, cholinergic neurons, and acetylcholine injury are also play important roles in AD. Symptomatic treatment for AD patients is the future trend of treatment.

Modern pharmacology and drug development usually follow a single objective principle, which may have led to the failure of most anti-AD compounds in clinical trials. The advantages of natural drugs are mild curative effect and few side effects, which have become a hot spot for AD treatment. As described above, according to the five pathogenesis hypotheses of AD, many effective natural products have been identified to be able to alleviate the symptoms of AD according to different targets and mechanisms. Although some drugs have entered clinical trials, most are still in the subclinical trial phase, and further evidences are needed to provide the efficacy of these drugs in clinically relevant animal models of AD to find appropriate doses and toxicity etc. Similarly, the pharmaceutical manufacturing industry, including the identification of suitable methods for extracting active ingredients, is equally crucial. In addition, it is still unclear whether they can exhibit or limit drug resistance due to their multi-target properties. In summary, the therapeutic advantages of natural medicines for AD have surpassed existing single-targeted drugs. In order to complete the laboratory-to-clinical conversion, related pharmacology, toxicology, extraction process, and combined use with other drugs need further research and confirmation.

## Author Contributions

YM, Ma-wY, X-wL, J-wY, J-zC, and Me-wY wrote the paper. XH, L-lZ contributed to paper revision. F-fH and S-lY are responsible for writing promotion in grammars and languages, the idea, and fund.

## Conflict of Interest

The authors declare that the research was conducted in the absence of any commercial or financial relationships that could be construed as a potential conflict of interest.
